# Multi-Channel Interactive Reinforcement Learning for Sequential Tasks

**DOI:** 10.3389/frobt.2020.00097

**Published:** 2020-09-24

**Authors:** Dorothea Koert, Maximilian Kircher, Vildan Salikutluk, Carlo D'Eramo, Jan Peters

**Affiliations:** ^1^Intelligent Autonomous Systems Group, Department of Computer Science, Technische Universität Darmstadt, Darmstadt, Germany; ^2^Center for Cognitive Science, Technische Universität Darmstadt, Darmstadt, Germany; ^3^Models of Higher Cognition Group, Department of Psychology, Technische Universität Darmstadt, Darmstadt, Germany; ^4^Robot Learning Group, Max Planck Institute for Intelligent Systems, Tübingen, Germany

**Keywords:** human-robot interaction, interactive reinforcement learning, human-centered AI, robotic tasks, user studies

## Abstract

The ability to learn new tasks by sequencing already known skills is an important requirement for future robots. Reinforcement learning is a powerful tool for this as it allows for a robot to learn and improve on how to combine skills for sequential tasks. However, in real robotic applications, the cost of sample collection and exploration prevent the application of reinforcement learning for a variety of tasks. To overcome these limitations, human input during reinforcement can be beneficial to speed up learning, guide the exploration and prevent the choice of disastrous actions. Nevertheless, there is a lack of experimental evaluations of multi-channel interactive reinforcement learning systems solving robotic tasks with input from inexperienced human users, in particular for cases where human input might be partially wrong. Therefore, in this paper, we present an approach that incorporates multiple human input channels for interactive reinforcement learning in a unified framework and evaluate it on two robotic tasks with 20 inexperienced human subjects. To enable the robot to also handle potentially incorrect human input we incorporate a novel concept for self-confidence, which allows the robot to question human input after an initial learning phase. The second robotic task is specifically designed to investigate if this self-confidence can enable the robot to achieve learning progress even if the human input is partially incorrect. Further, we evaluate how humans react to suggestions of the robot, once the robot notices human input might be wrong. Our experimental evaluations show that our approach can successfully incorporate human input to accelerate the learning process in both robotic tasks even if it is partially wrong. However, not all humans were willing to accept the robot's suggestions or its questioning of their input, particularly if they do not understand the learning process and the reasons behind the robot's suggestions. We believe that the findings from this experimental evaluation can be beneficial for the future design of algorithms and interfaces of interactive reinforcement learning systems used by inexperienced users.

## 1. Introduction

Future robots are expected to cope with a variety of different tasks which renders manual programming of each task highly difficult. One promising approach for acquiring new skills from non-expert users is to learn skill libraries from demonstrations (Pastor et al., 2009; Koert et al., 2018). However, even if a robot has already learned a skill library it remains a challenge to learn how to sequence such skills correctly to perform more complex tasks. In such cases, Reinforcement Learning (RL) provides a way for a robot to learn from experience while observing the effects of the chosen actions, i.e., the applied skills, on the environment (Kober et al., 2013; Sutton and Barto, 2018). The main challenges in RL are related to the uncertainty that the agent has about the environment it is interacting with, which is usually modeled as a Markov Decision Process (MDP). Since the agent usually cannot access the model of this MDP, there is a need to explore the states of the MDP sufficiently well, in order to understand which actions are appropriate to take in which states. This would not be an issue if the cost of collecting samples was not involved. Especially in real robotic applications, the cost and required time for collecting samples can be a limiting factor. Additionally, in real robotic scenarios, there might be high costs assigned to taking wrong actions, such as breaking valuable objects, the robot's hardware, or even cause harm to humans. This can further confine the exploration of the agent. One possible way to tackle these problems, speed up the learning, and make RL applicable for more robotic tasks is to provide humans a possibility to interact with the robot during the RL learning process. On the one hand, such interaction provides potentially helpful input and advice. On the other hand, it also introduces the need to consider human preferences and the challenge to cope with potentially wrong input and suggestions of the human. The use of human feedback in RL is thereby not new and was already successfully applied, for instance, to provide demonstrations of good behavior (Argall et al., 2008), to provide a supplementary reward (Knox and Stone, 2011), or to manipulate the available actions (Cruz et al., 2016). Human guidance can be particularly helpful when a RL agent does not have access to any background knowledge that a human has, but has to learn solely based on environmental feedback.

However, many of the results involving interactive RL algorithms in the literature were obtained in simulation environments (Thomaz and Breazeal, 2008; Knox and Stone, 2011; Li et al., 2016; Peng et al., 2016). There is a lack of experimental studies for interactions of inexperienced users with RL systems on real robotic tasks (Suay and Chernova, 2011; Knox et al., 2013), in particular under the assumption that human input might be partially incorrect. Therefore, in this paper, we present a novel interactive RL framework that integrates multiple channels for human input, namely action suggestion, and prohibition as well as feedback and sub-goal definition. We evaluate this approach for two robotic tasks with non-expert users. While our definition of different input channels is similar to the approach in Suay and Chernova (2011), we additionally incorporate the concept of self-confidence that allows the robot to question potentially wrong human input after an initial training phase.

Overall, the main contribution of this paper, therefore, is the evaluation of our multi-channel interactive RL framework, which includes our concept for self-confidence of the robot, on two sequential robotic tasks with 20 inexperienced human subjects. The second robotic task is specifically designed to investigate the effects of partially wrong human input on the learning process. The concept of self-confidence enables the robot to achieve learning progress even if human input is incorrect. Moreover, we evaluate how humans react to the robot's suggestions when it considers their input to be incorrect as well as which input channels are preferred by humans. We consider this evaluation important for the successful design of future interactive robotic RL algorithms and the corresponding interfaces for inexperienced humans to use.

The rest of the paper is structured as follows. Section 2 summarizes related work. Section 3 introduces our framework for multi-channel interactive RL and the concept of self-confidence of the robot. In section 4, we evaluate the proposed approach on two robotic tasks, report our results from the experiments with human users, and discuss the results and limitations. Lastly, section 5 concludes the paper and gives an outlook on possible directions for future work.

## 2. Related Work

Different forms of human input to Reinforcement Learning have been proposed in the literature. We first give a brief overview of these approaches, where we mainly focus on the use of human feedback and human action advice in RL algorithms. Subsequently, we discuss related work that integrates multiple human input channels and existing evaluations of such approaches on robotic tasks with human subjects.

As one of the first approaches, inspired by animal clicker training, human feedback has been used as a reward signal in Reinforcement Learning with a robotic dog by Kaplan et al. (2002) and animated characters by Blumberg et al. (2002) to reinforce desired behaviors. The use of object and action related feedback in a sequential kitchen task was evaluated by Thomaz et al. (2005), in which users interactively trained an agent in a simulated kitchen environment. Knox and Stone (2008) introduced the TAMER framework to learn a human reward model from infrequent feedback and extended their approach for combining the learned human feedback model with environmental rewards in Knox and Stone (2010). Knox and Stone (2011) discuss different ways of combining a model learned from human input with RL, namely reward shaping, Q-augmentation, control sharing, and action biasing. Our concept for self-confidence matches the definition of their introduced combination parameters in the control sharing approach. However, Knox and Stone (2011) did not use these combination parameters to allow the robot to question human input but only for the action selection mechanism.

Judah et al. (2010) proposed to iterate between practice sessions and sessions where users can label trajectory data with good or bad feedback. This can be useful in situations where, e.g., real-time feedback is impossible due to speed or when demonstrations are hard to provide due to difficult control. However, this is not the case for the type of tasks we consider in this paper. Griffith et al. (2013) introduced the ADVISE algorithm which uses policy shaping to incorporate human feedback and treats human feedback not as an evaluative reward, but as a label on the optimality of the policy. Cruz et al. (2018b) used multimodal audio-visual input commands along with confidence values to indicate the trustworthiness of the given feedback. Moreover, the use of implicit feedback signals, such as models of user's affective states or measures for their valence or task engagement, have been proposed in the literature (Leite et al., 2011; Gordon et al., 2016; Ritschel et al., 2017; Tsiakas et al., 2018).

Besides the use of human feedback signals also action advice during the RL process was explored in the literature. Maclin and Shavlik (1996) were one of the first to include human action suggestions in reinforcement learners. Wang et al. (2003) used such action suggestions and subgoal suggestions to bias the reward in robot navigation tasks. Kuhlmann et al. (2004) presented a framework with which a soccer robot can learn from advice in the form of natural language commands which were combined with a RL function approximator. Argall et al. (2008) introduced advice operators for continuous state and action spaces and evaluated them on a segway positioning task where post-execution feedback was used as demonstration data. Moreno et al. (2004) introduced the concept of supervised RL in which multiple advice sources are combined with the learned policy and a decision module to choose which actions to take. They introduced credit assignment for different advice sources, which relates to our concept of self-confidence. But in contrast to their approach, we use a shared control strategy for the selection between human advice and the robot's policy and increase the self-confidence only after an initial training phase. Moreover, Moreno et al. (2004) did not evaluate their concept for credit assignment in interaction with humans. Supervision of the robot's action suggestion has been investigated to transfer technical and social skills to a robot in the context of an educational task (Senft et al., 2019). There was also an investigation of how agents can advise other RL agents about action suggestions (Torrey and Taylor, 2012, 2013). This might not directly correspond to human advice but it provides valuable insights to beneficial advice strategies. Additionally, it has been linked to the concept of affordances to reduce the state and action space during exploration (Cruz et al., 2016). Torrey and Taylor (2013) report that different ways of advising, such as early, importance, mistake correcting, and predictive advising may become beneficial when teaching on a budget. Cruz et al. (2018a) studied which types of advisors are most beneficial during learning of the agent in a simulated domestic scenario.

Another line of work is to consider human prior knowledge of task decomposition to achieve a form of curriculum learning for more complex tasks (Wang et al., 2020). Human input to RL has also been used in combination with policy search methods and to improve robot skills on a trajectory level (Celemin and Ruiz-del Solar, 2016, 2019; Celemin et al., 2019). This is also very relevant for robotic applications, however, it should be noted that in this paper we focus only on the sequencing of skills as high-level actions.

The combination of multiple human inputs in RL algorithms was proposed before, e.g., to inform the reward structure (Wiewiora et al., 2003). Such a combination was also used for initially unknown spoken word commands for positive or negative feedback and action suggestion for a robotic pick and place task (Grizou et al., 2013). Further, Abel et al. (2017) utilized protocol programs as an intermediate layer between RL-agent and environment to combine different RL algorithms with varying forms of human feedback. While this approach of Abel et al. (2017) is highly related to our work in terms of incorporating different inputs in a modular structure, it was not evaluated on real robotic tasks with human users but only in simulated environments, such as grid world and pong. Our combination of channels for feedback and action advice also relates to the approach of Suay and Chernova (2011), but additionally incorporates the concept of self-confidence to allow the robot to question human input.

Overall, only a few studies exist about applications of interactive RL frameworks on real robotic applications (Suay and Chernova, 2011; Knox et al., 2013) and their evaluations with inexperienced human users. Human users can significantly differ from simulated oracles and studies with real subjects, therefore, provide valuable insights into actual human behaviors. Isbell et al. (2006) reported that in environments with humans many RL assumptions, e.g., on the reward can be violated due to drift or bias. Thomaz and Breazeal (2006) conducted a user study with a simulated kitchen task where they reported that humans also want to provide a future reward to guide the robot. They also found that humans used object-specific rewards to guide the robot even though it was conceived as feedback. Thomaz and Breazeal (2008) reported that users might use feedback as a motivation signal and that humans may change their reward signal when the agent is learning. Judah et al. (2010) showed that humans might get annoyed if a RL-algorithm does not directly respond to their feedback. Further, Loftin et al. (2014) stated that there is a need to learn models of human feedback strategies and suggested designing RL-agents to understand and adapt to different users' training strategies.

However, these prior studies consider only tasks, in which humans generally only gave useful and correct input. To the best of the authors' knowledge, there is no related work on applications of interactive RL for robotic tasks that were specifically designed to include incorrect human feedback and in which the robot starts to actively question the human input during the learning process.

## 3. Multi-Channel Interactive Reinforcement Learning

In this section, we present our approach to integrate multiple channels for human input into a framework for interactive RL. We hereby focus on learning sequential tasks. This refers to learning how to sequence high-level actions which can, e.g., represent skills in the form of motion primitives that the robot already learned before. In a RL setting an agent, which is the robot in our case, interacts with the environment and tries to learn a policy π to decide which action *a* it should choose in a state *s* to maximize its accumulated rewards. A reward *r* is given after each transition into a next state *s*′ and the rewards are accumulated over an episode of a finite number of time steps *t*. While in the classical RL setting the robot learns independently, in interactive RL the robot may also incorporate human input during the learning process.

**Figure 1:**
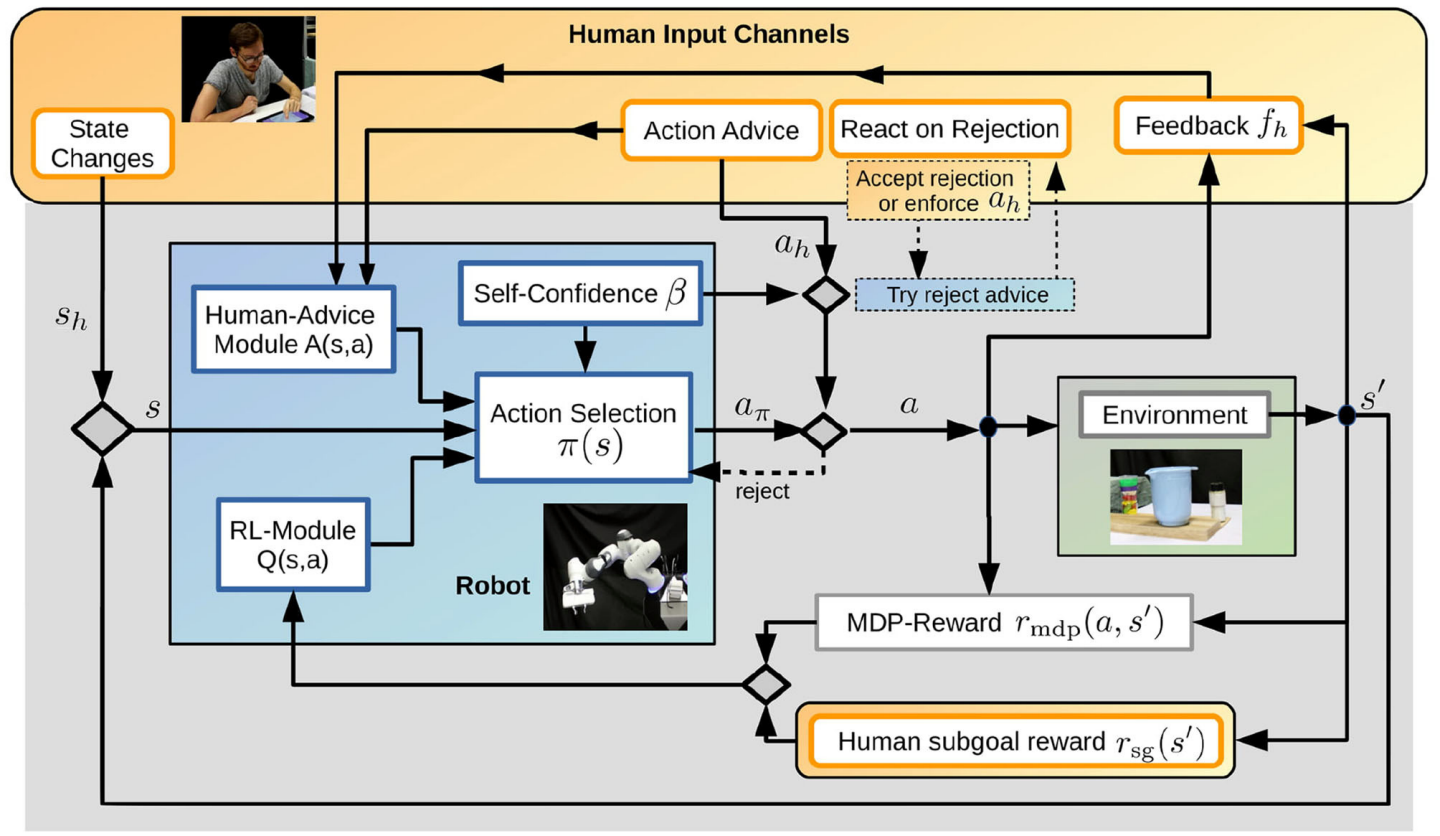
We propose an approach that integrates multiple human input channels (orange) in an interactive RL framework for sequential tasks. The human can prevent or suggest actions, give feedback after action execution, modify the state, or define subgoal rewards. Through interaction with the environment and the human input, the robot (blue) learns a policy that tries to maximize the overall reward and chooses actions based on the Human-Advice Module, the RL-module, and its self-confidence. The self-confidence also eventually enables the robot to question human action advice if it contradicts the robot's policy.

We first discuss the relevant channels for human input during RL of sequential robotic tasks in section 3.1. Afterwards, we present the different components of our multi-channel interactive RL framework, namely the RL-module in section 3.2, the human-advice module in section 3.3, the Action Selection mechanism in section 3.4 and our novel concept of self-confidence of the robot in section 3.5. Figure 1 shows an overview of the approach and Algorithm 1 summarizes the different steps during learning, where *i* refers to the episode index. In section 3.6 we provide details on the current implementation for the components of the framework, which was used for the experimental evaluations in this paper.

### 3.1. Human Input Channels

We use human input as a valuable source of information to guide the exploration of a robot in a RL setting, speed up the learning, and prevent disasters which could be caused by exploratory actions in real robotic scenarios. In our approach, we, therefore, consider multiple input channels, such as action suggestions or prevention of action executions, feedback after the execution of an action, subgoal reward definition, or state modifications by the human user.

#### 3.1.1. Human Advice on Planned Actions

Our framework allows the human to give specific advice on the next planned action of the robot. This input channel can be used before the actual execution of an action by the robot. Therefore, the robot first communicates the planned action according to its policy *a*_π_ to the human. The human then has two options to react to the proposed action with action advice *a*_h_. First, the human can simply reject the proposed action which is represented by *a*_h_ = −1. In case of such a rejection, the robot in the next step might propose another action according to its updated policy. The second option for the human is to suggest an alternative action to the robot which is indicated by *a*_h_ ≥ 0. This action then overwrites the proposed action in a supervised RL-fashion. If there is no input from the human on the proposed action, i.e., *a*_h_ = ∅ the robot just executes the proposed action. To summarize, the action *a* to be executed in the next step is chosen as

(1) $$\begin{array}{l} a=\{\begin{array}{l} \emptyset \;\text{if}\;a_{\text{h}}==-1\\ a_{\text{h}}\;\text{if}\;a_{\text{h}}\geq 0\\ a_{\pi }\;\text{if}\;a_{\text{h}}==\emptyset \end{array} \end{array}$$

The option of rejecting actions is particularly important to prevent disaster actions in real scenarios. We assume that this option can be used even if the human has no idea what the optimal action would be, but still recognizes potentially disastrous actions. The active suggestion of actions by the human can be used in tasks where the human knows how to guide the exploration of the robot.

#### 3.1.2. Human Feedback After Action Execution

After the execution of an action *a* the human can provide feedback *f*_h_ for this action. For our experiments we consider three options for feedback that is “Good,” “Bad,” or “I don't know” represented by

(2) $$\begin{array}{r} f_{\text{h}}\left(s,a\right)=\{\begin{array}{r} 1\\ -1\\ 0 \end{array} \end{array}$$

If no feedback is provided by the human this is also counted as neutral feedback *f*_h_(*s, a*) = 0. The option to provide feedback after action execution can be beneficial in tasks in which the human is not able to foresee the effects of choosing a specific action but is able to judge it afterwards.

#### 3.1.3. Human Subgoal Rewards

Action advice and feedback after action execution are incorporated online during learning. Additionally, we provide an input channel to define subgoal rewards before the learning phase for the task starts. These subgoal rewards reward the occurrence of certain states and are limited to a discrete state space in the current formulation. A subgoal reward $r_{\text{sg}}^{i}\left(s\right)$ is hereby defined for part of the state vector *s* = {*s*_0_, .., *s*_*d*_, ..*s*_*D*_}

(3) $$\begin{array}{l} \begin{array}{l} r_{\text{sg}}^{i}(s)=\{\begin{array}{l} 1\text{ if, }s==s_{\text{sg}}\\ 0\text{ else} \end{array} \end{array} \end{array}$$

and the human may define multiple subgoal rewards. The final subgoal reward definition is given by

(4) $$\begin{array}{l} r_{\text{sg}}\left(s\right)=\underset{i}{\sum }r_{\text{sg}}^{i}\left(s\right). \end{array}$$

All human-defined rewards are constant throughout the learning, thus need to be specified for a task *a priori* by the user. We, therefore, consider this channel most useful in tasks where a human is sure about the subgoals before task execution. In the current version, our framework does not handle cases in which the human could change subgoals because they notice during the interaction that the originally defined subgoals were wrong. Therefore, the definition of subgoals requires a good understanding of the task by the human which can be a limiting factor in more complex tasks.

#### 3.1.4. Human State Modifications

In many situations, it might also be helpful for the robot's learning to make use of the option that a human can physically modify the state of the environment. Hereby, the next state *s*′ can be changed by human interference with the environment. This can be used, e.g., to undo the effects of an action, to let the robot experience the same situation again, or to have a reset to a specific state after the end of an episode. The environmental state modification by the human is modeled as

(5) $$\begin{array}{l} {\left(s\right)}^{t+1}=\{\begin{array}{l} {\left(s^{\prime }\right)}^{t}\;\text{if}\;s_{\text{h}}==\emptyset \\ s_{\text{h}}\;\text{else,} \end{array} \end{array}$$

where *t* denotes a single step of an episode and *s*_h_ is the environmental state after the modification by the human. In particular, such modifications can help the agent to reach states that otherwise would take longer to be reached through pure exploration and help the agent to gather experience in these states. State modifications can also enable the human to let the agent visit important states more frequently.

### 3.2. Reinforcement Learning Module

Whenever the robot takes an action *a* in a state *s* it transits to the next state *s*′ and receives a reward that consists of the environmental reward *r*_mdp_ and the human subgoal reward *r*_sg_, which was introduced in section 3.1.3. The goal of the robot is to maximize the combined reward

(6) $$\begin{array}{l} r\left(s,a\right)=r_{\text{mdp}}\left(s,a\right)+\eta {\;r}_{\text{sg}}\left(s,a\right), \end{array}$$

where η is a factor to scale the influence of human subgoal rewards which needs to be handtuned in our current version of the framework. The robot therefore uses the samples *s, a, s*′, *r* to learn a policy π(*s*) that maximizes the total reward.

The Reinforcement Learning module hereby learns a Q-function *Q*(*s, a*), that is the cumulative reward for starting in *s*, applying action *a*, and in the resulting state *s*′, act optimally.

### 3.3. Human-Advice-Module

For our approach, we use human input during learning in two ways. On the one hand, direct action suggestion influences the exploration which might change the next performed action by the robot. This is comparable to a supervised RL setting (Moreno et al., 2004). On the other hand, since human input might be sparse and the human might not repeatedly give input in the same states, we also learn a model of human feedback and advice, such that this model can be used to guide the agent even if no human input is given. This is comparable to the approach of learning a model for human reward as proposed in Knox and Stone (2008), even though their model is solely based on human feedback as an input.

In our framework, the human advice module, therefore, learns a function *A*(*s, a*) that indicates which action is the human would most likely suggest or provide positive feedback on. Since we assume that both human feedback and advice would try to guide the robot in the same way, we learn a joint human advice module from feedback and advice.

### 3.4. Action Selection

Based on the RL module and the human advice module the robot decides in each step which action *a*_π_ to present to the human for potential advice. The literature contains different comparisons of how to combine a RL-based Q-function and a human advice module. In Knox and Stone (2011) it is discussed that methods that act on the action selection rather than change the Q-function directly generally work better and outperform other combination methods. Following this argumentation, we believe it is beneficial to use a control sharing approach for action selection in our framework.

The robot hereby follows the human advice module with the probability 1 − β, if the advice module is not indifferent about all actions in the state

(7) $$\begin{array}{l} \text{if not}\;A\left(s,a_{j}\right)==\underset{a}{\max}\left(A\left(s,a\right)\right)\;\forall \;a_{j},\\ \text{with probability }1-\beta ,\;a_{\pi }=\arg\underset{a}{\max}\left[A\left(s,a\right)\right],\\ \text{with probability }\beta ,{\;a}_{\pi }\text{ according to policy based on}\\ \text{ RL-module,} \end{array}$$

where β denotes the self-confidence of the robot. Our concept of this self-confidence is explained in detail in section 3.5. Alternatively, with probability β, the robot follows a policy based on the Q-function of the RL module.

### 3.5. Self-Confidence

If the human understands the task at hand well and provides useful input, human input can speed up the learning of the robot. However, incorrect human input can also slow down or even prohibit learning of the task. Therefore, we introduce the concept of self-confidence β of the robot into our interactive RL framework. First, this self-confidence is used as a combination parameter of the RL and the human advice module, as described in section 3.4. Second, the self-confidence can also be used by the robot to question an action suggested by the human if it contradicts the robot's learned Q-function. This can be expressed by the probability of trying to reject a human suggestion *p*(reject *a*_h_) = β. Such a rejection is implemented in our framework as a feedback message to the human whenever the robot considers the human action input to not be beneficial. However, it still leaves the freedom of choice to the human such that they can decide whether to execute the originally advised action regardless or rather follow an alternative suggestion based on the robot's Q-function.

At the beginning of the learning process, the robot has no own experience, which is represented by a low self-confidence, e.g., β = 0. Due to that, it will follow all suggestions given by the human or the human advice module and always assume human input to be beneficial and correct. However, while the robot learns from its own experience it will slowly converge toward a more informative Q-function and can eventually distinguish between good and bad human advice. With this, the self-confidence can eventually increase during learning, allowing the robot to question and deal with potentially incorrect human input. As such, the self-confidence needs to be computed such that it provides the robot a notion of the quality and convergence of its learned policy, which is represented by the Q-Function of the RL-module. In particular, the self-confidence can vary for different states and should relate the robot's trust in its own policy with its trust in the human input.

**Algorithm 1:** MINT-RL.

1: init *Q, A*, e.g., tabular as ***Q***[*s, a*] = 0 and ***A***[*s, a*] = 0 ∀*s, a*
2: init visits per state ***v*** = 0 ∀*s*,
3: init β = 0, *s* = *s*_0_, *i* = 0
4: **while** i < Maximum Episodes **do**
5: ***v***[*s*] = ***v***[*s*]+1
6: Chose *a*_π_ from action selection policy π(*s*, ***Q***, ***A***, β, ***v***), e.g., Shared Control with ε-greedy Algorithm 4
7: present *a*_π_ to human
8: *a*_h_ ← human action advice
9: **if** *a*_h_ = =∅ **then**
10: *a* = *a*_π_
11: **else**
12: **if** *a*_h_ not optimal according to *Q*(*s, a*) **then**
13: *p* = random sample from uniform distribution
14: **if** *p* < β **then**
15: suggest human to reject *a*_h_
16: **if** human accepts rejection **then**
17: *a* = *a*_π_
18: **else**
19: *a* = *a*_h_
20: **else**
21: *a* = *a*_π_
22: **else**
23: *a* = *a*_h_
24: *s*' ← execute *a* in *s*
25: $r\leftarrow r_{\text{mdp}}\left(a,s^{\prime }\right)+r_{\text{sg}}\left(a,s^{\prime }\right)$
26: *f*_h_ ← human feedback
27: update *Q* from *r, s, a, s*′, e.g., Tabular Q-Learning Algorithm 2
28: update *A* from *s, a*_π_, *a*_*h*_, *f*_*h*_, e.g., Tabular Human-Advice-Module Algorithm 3
29: **if** human changes the state **then**
30: *s* = *s*_h_
31: **else**
32: *s* = *s*′
33: update β, e.g., with const linear increase Algorithm 5
34: i++

### 3.6. Component Implementation

This subsection presents the implementation that was chosen for the single components of the framework in the experimental evaluation for this paper. While for now the chosen implementations follow rather simplistic approaches and are tailored for our experimental setting the modularity of the framework allows easy replacements with more complex implementations of single components for future applications.

#### 3.6.1. Tabular Q-Learning Reinforcement Learning Module

In the experiments in this paper, we use tabular Q-Learning as a RL-algorithm. The Q-function is hereby represented by a table with *S* × *A* entries, where *S* is the total number of states and *A* the total number of actions. This table is initialized with zeros. In Q-Learning (Watkins, 1989) for each sample < *s, a, s*′, *r* > the Q-function is updated according to

(8) $$\begin{array}{l} Q\left(s,a\right)=Q\left(s,a\right)+\alpha \left(s\right)\left(r\left(s,a\right)+\gamma \;\underset{a}{\max}Q\left(s^{\prime },a\right)-Q\left(s,a\right)\right), \end{array}$$

where α(*s*) is the learning rate in state *s* and γ is the discount factor. We chose here to decrease the learning rate over time dependent on the number of visits *v*(*s*) of a state that is α(*s*) = 1/*v*(*s*), as this is a common practice in the literature. Therefore, we initialize a vector ***v*** of length *S* with zeros and update it whenever a state is visited. Algorithm 2 summarizes the update procedure of the Q-function for each sample.

**Algorithm 2:** Tabular Q-Learning Update.

1: input: *r, s, a, s*′, γ, ***v***, ***Q***
2:
3: α = 1/***v***[*s*]
4: $Q\left[s,a\right]=Q\left[s,a\right]+\alpha \left(r+\gamma \;\underset{a^{\prime }}{\max}Q\left[s^{\prime },a^{\prime }\right]-Q\left[s,a\right]\right)$

For future applications, this implementation of the RL-module could be replaced by another off-policy RL-algorithm, if desired.

#### 3.6.2. Tabular Human Advice Module

For simplicity of the evaluations, in this paper, we represent the human advice module as a tabular function *A*(*s, a*), which we initialize with zeros for all state-action pairs. This tabular function is updated whenever human advice or feedback is received. In particular, when the human suggests an action *a*_*h*_ in a state *s* we increase the value of *A*(*s, a*_*h*_) and if the human rejects a suggested action *a*_π_ (indicated by *a*_*h*_ = = −1) we decrease the value of *A*(*s, a*_π_). For human feedback which follows after an action *a* was performed in state *s*, we increase or decrease the values of *A*(*s, a*) accordingly. The implementation for the update of the human advice module is summarized in Algorithm 3.

It should be noted that this simplistic view on the human advice module can be easily exchanged by any more complex model, which learns a similar function from human advice and feedback.

**Algorithm 3:** Tabular Human Advice Module.

1: input: *s, a*_π_, *a*_*h*_, *a, f*_*h*_, ***A***
2: **if** *a*_*h*_ not ∅ **then**
3: **if** *a*_*h*_ = = − 1 **then**
4: ***A***[*s, a*_π_] = ***A***[*s, a*_π_] − 1
5: **else**
6: ***A***[*s, a*_*h*_] = ***A***[*s, a*_*h*_] + 1
7: ***A***[*s, a*] = ***A***[*s, a*] + *f*_*h*_

#### 3.6.3. Shared Control With ε-Greedy Policy for Action Selection

We implemented a shared control approach between the human advice model and an ε-greedy policy based on the RL module. The robot hereby follows the human advice module with a probability of 1 − β, if the human advice module is not indifferent about all actions in the state, as described in section 3.4. Alternatively, the robot follows an ε-greedy policy based on the Q-function of the RL module. Thereby, it selects a random action with a probability of ε(*s*) and otherwise chooses the action according to the maximum of the Q-function as

(9) $$\begin{array}{l} a_{\pi }=\arg\underset{a}{\max}\left[Q\left(s,a\right)\right] \end{array}$$

Hereby we decrease ε(*s*) based on the number of visits *v*_*s*_ of a state according to $\varepsilon \left(s\right)=1/\sqrt{v_{s}}$, which is a common choice in the literature. However, since in interactive RL the number of learning steps can be much lower than in classical RL we think in future work different forms of computation should be investigated.

In case the human advice module gives equally good advice for more than one action we follow an ε-greedy policy with the RL module on this subset of actions. Algorithm 4 summarizes this policy.

**Algorithm 4:** Shared Control with ε-greedy Policy.

1: input: *s*, ***Q***, ***A***, β, ***v***, **ε**
2: $\varepsilon =1/\sqrt{v\left[s\right]}$
3: *p*_1_ = random sample from uniform distribution
4: **if** *p*_1_ < = (1 − β) **then**
5: $a_{A}=\underset{a}{\arg\max}$ ***A***(*s, a*)
6: **if** len(***a***_*A*_) == 1 **then**
7: *a*_π_ = ***a***_*A*_
8: **else**
9: *p*_2_ = random sample from uniform distribution
10: **if** *p*_2_ < ε **then**
11: *a*_π_ = random choice from ***a***_*A*_
12: **else**
13: $a_{A,Q}=\underset{a}{\arg\max}$ ***Q***(*s, a*) ∀*a* in ***a***_*A*_
14: *a*_π_ = random choice from ***a***_*A, Q*_
15: **else**
16: *p*_3_ = random sample from uniform distribution
17: **if** *p*_3_ < **ε** **then**
18: *a*_π_= random choice between all actions
19: **else**
20: $a_{Q}=\underset{a}{\arg\max}$ ***Q***(*s, a*)
21: *a*_π_ = random choice from ***a***_*Q*_

#### 3.6.4. Heuristic Increase for Self-Confidence

In general, the increase of the self-confidence should resemble the convergence of the learning process of the RL-module. The choice of a good theoretical model for such an increase is not straightforward. Due to that, in our experiments, we tailored the increase of the self-confidence for the chosen problems and constantly increased the self-confidence by a predefined factor after a defined number of initial training episodes *I*_min_. Algorithm 5 summarizes this heuristic increase. Even if the current implementation does not represent an actual self-confidence of the robot in the correctness of its policy, being able to question the human' inputs can provide valuable insights to human reactions to the general concept of robotic self-confidence.

**Algorithm 5:** Increase Self-Confidence Heuristic.

1: input *i* and β
2: **if** *i* > *I*_min_ **then**
3: β = min{β + δ_β_, 1}

## 4. Experimental Evaluation

**Figure 2:**
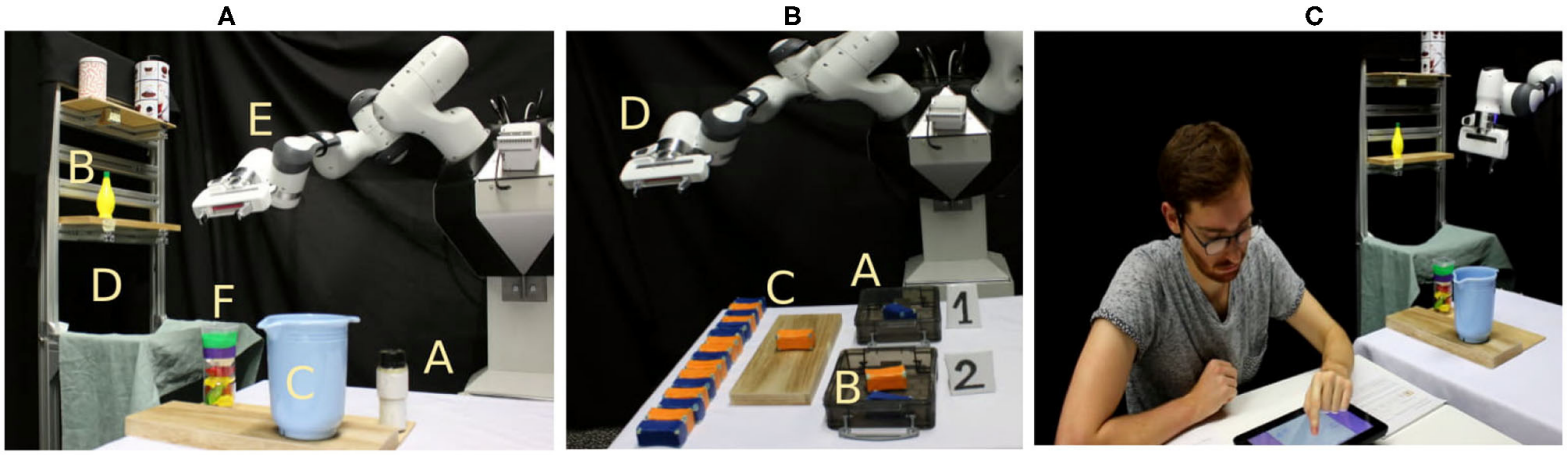
We evaluate the proposed framework on two sequential robotic tasks. **(A)** In the first task, the robot should learn how to finish a cocktail by pouring sugar (A) and lemon-juice (B) into a bowl (C). At the start of an episode, ingredients are either at the shelf (D) or close to the bowl and the robot starts in his start position (E). An episode fails if the robot pours ingredients onto the floor or adds the ingredient chocolate (F) to the bowl. **(B)** In the second task, the robot should learn to sort objects according to their weight into two boxes (A,B). However, this sorting criterion is not known to the human such that they might first think they should be sorted by color, which results in partially incorrect human input. In each episode, one object (C) is presented to the robot that starts at his start position (D) and the episode ends after the object was sorted in a box or unsuccessfully dropped at the start point. **(C)** We evaluate both tasks with 20 inexperienced human subjects that interact with the robot over a graphical user interface.

In this section, we evaluate our approach on two sequential robotic tasks (shown in Figure 2) with both, simulated and real human input from experimental evaluations with 20 users (12 male, 8 female). Regarding their age groups, two participants were between 18 and 20 years, 10 were between 21 and 29 years, 5 were between 30 and 39 years and three were between 50 and 59 years old. In addition, the participants were mostly inexperienced with robots, which means 11 of the participants reported never having interacted with a robot before our study, four having one other encounter before our study, another four having one to ten encounters and only one having more than 20 encounters with a robot before the study. Concerning the obtained results, we want to point out that 20 subjects are only a small sample size and we believe the results can therefore only indicate trends and tendencies.

In the experiments with simulated human feedback, we show the principle influence of different input channels on the tasks. In the first robotic task, we evaluate how the real human subjects use and understand the input channels, and which types of input they prefer. In the second robotic task, we additionally investigate how humans react to the concept of self-confidence of the robot and how they respond if a robot starts to make own suggestions, once it recognizes human input might be incorrect.

In the following, we report results for both tasks with simulated human input and subsequently the findings from the conducted human experiments. Figure 5A summarizes the procedure for the experiments with real human input, which is also explained in detail in sections 4.1.2 and 4.2.2. In the following, all statistical tests are performed on a significance level of α_s_ = 0.05. For the Mann-Whitney-*U* tests, we report the test statistic *U*, the standardized statistic *z*, the *p*-value, and the sample sizes *n*_1_ and *n*_2_. For the Wilcoxon signed-rank test we report the test statistic *T*, the standardized statistic *z*, the *p*-value, and the resulting sample size *n* after removing samples with zero differences.

### 4.1. Robotic Kitchen Task

In the first robotic task, we evaluate the influence of human input during interactive RL when it can be assumed that human input is mainly correct and the human has sufficient prior knowledge on how the task should be solved. The task is inspired by Sophie's kitchen task from Thomaz et al. (2005). Since the focus in the evaluation of this task is on the comparison of the human input channels we did not use the concept of self-confidence, assumed input to be always correct in the simulation and disabled the option for the robot to question human input.

In our kitchen task, the robot should learn to add specific ingredients (that are known to the human) to a bowl in order to complete a cocktail. At the beginning of the task, all ingredients can be either on the shelf or close to the bowl. At least one ingredient is at each of those locations and the robot starts at his start position as depicted in Figure 2. The state space of the corresponding MDP is formally defined by the position of the arm, which can be either AT-BOWL, AT-HOME, or AT-SHELF, the positions of the objects, which can be AT-SHELF, CLOSE-TO-BOWL, or IN-ARM and the state of the bowl which is defined by the contained ingredients. A bowl state where object 1 was added to the bowl but objects 2 and 3 are not would be represented by < 1, 0, 0 >. This definition of the state space with *N* objects results in 3 * 3^*N*^ * 2^*N*^ states. The actions are defined as GO-TO-BOWL, GO-TO-SHELF, GRASP-OBJ-X, POUR, and PUT-DOWN, which results in 4+N actions. Not all actions are available in all states, e.g., the robot can only pour or put something down if it grasped an object before, and it can only grasp objects at the location where it currently is, e.g., at the shelf or close to the bowl. An episode is successfully finished if the bowl contains the desired ingredients. An episode ends unsuccessfully if ingredients are poured to the floor, i.e., choosing the pouring action when the arm is not at the bowl, or if wrong objects are poured into the bowl. The reward is defined as

$$\begin{array}{l} r=\{\begin{array}{l} 100\;\text{ if episode ends successfully}\\ -100\text{ if episode ends not successfully}\\ 0\text{ if episode ends due to reaching the maximum}\\ \text{ number of steps}\\ 0\text{ in all other states that do not end the episode} \end{array} \end{array}$$

In our experiments, we defined the missing ingredients for the cocktail as lemon-juice and sugar. Additionally, the ingredient chocolate is present in the setup. These three objects result in 648 states and seven actions. The task setup is shown in Figure 2A.

#### 4.1.1. Simulated Human Input

In this section, we evaluate the influence of the different input channels on the learning process of the robotic kitchen task using simulated human input.

**Figure 3:**
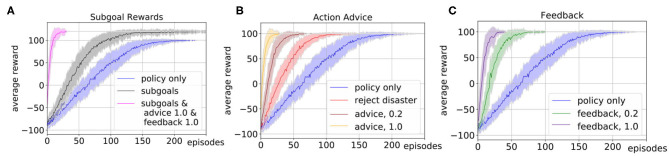
We evaluate the influence of different types of simulated human input on the learning process of the robotic kitchen task. The plots show the average reward over 50 experiments and 20 evaluation runs. We plot the mean (solid line) and standard deviation (shaded area). **(A)** Shows the influence of subgoal definition (gray) and the results with the combination of subgoals, feedback, and advice (pink). **(B)** Shows the influence of rejecting disaster actions (red) and correct action suggestions with probabilities 1.0 (orange) and 0.2 (brown) for action advice. **(C)** Shows the influence of correct feedback after action execution with probability 1.0 (purple) and 0.2 (green) in comparison to learning without human input (blue).

First, we evaluate the influence of the human subgoal definition, where we chose η = 10. The parameter η was hereby hand-tuned with respect to the overall task reward and does not claim to be an optimal choice. In particular, in tasks with a sparse reward structure subgoal rewards can help to guide the exploration and accelerate the learning of the robot. Our simulated human input defines subgoals whenever one of the ingredients sugar or lemon juice is added to the bowl such that the state of the bowl changes to contain part of the desired ingredients, and rewards this with +10. Figure 3A shows the comparison of the learning with subgoals (black) and without subgoals (blue). To obtain a mean value of the performance after each episode, we averaged the policy learned up to that point over 20 evaluations runs and repeated this for 50 experiments with different random seeds. The plot shows the mean and standard deviation of the average reward plotted over the number of episodes. It shows that subgoal definition results in a steeper learning curve.

Next, we evaluate the influence of different forms of action advice. The human oracle is hereby implemented in a way that it can reject actions that would lead to a disaster or advice actions that can be beneficial for the current state with a predefined probability. Figure 3B shows a comparison of the learning curve without action advice (blue), with rejection of actions (red) and action suggestions with probability 1.0 (orange) and 0.2 (brown). The results show that suggestions of correct actions can speed up the learning up to a factor of 10, but even if the suggestions are only provided with lower probability or if only the option of preventing disaster actions is used the learning can be accelerated.

In Figure 3C we show the results of the influence of feedback after action execution on the learning process. As in the advice, we assume the simulated feedback to always be correct and given with a certain probability. Again, the learning can be accelerated even if the feedback is not given in every state. The learning is hereby slightly slower than in the case of the advice since the feedback can not actively guide the exploration.

#### 4.1.2. Real Human Input

**Figure 4:**
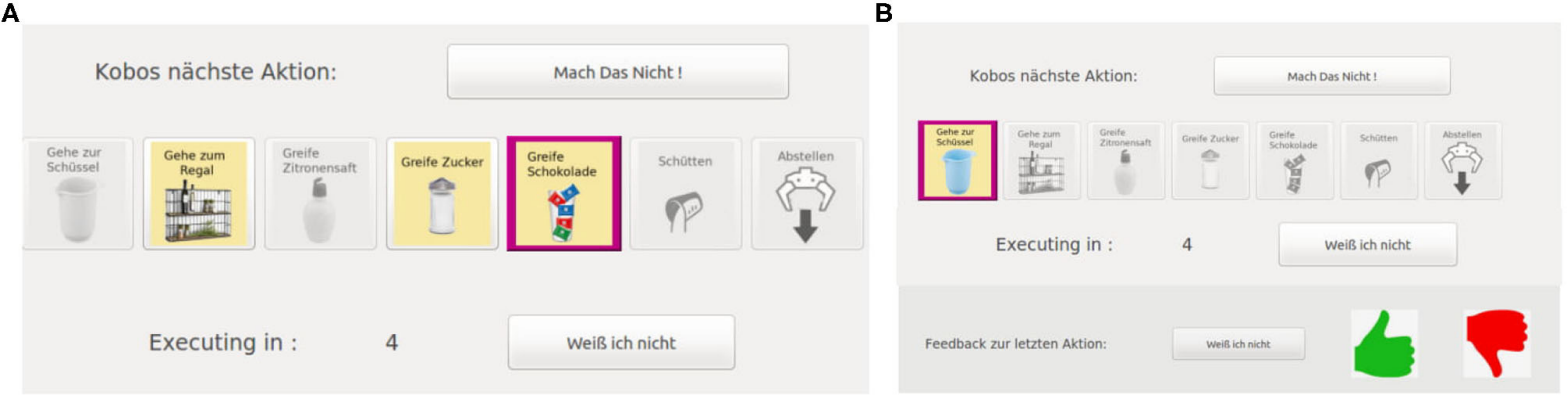
The subjects interact with the robot named Kobo through a graphical user interface on a touch display. The main parts of the interface are labeled in German since the study was conducted in Germany. Here, we show the interfaces for the different modes in the Kitchen Task. The interface for the sorting task only differed in the displayed actions. **(A)** In Mode A, a proposed action is shown to the subjects by highlighting it in pink (“Kobos nächste Action” — Kobo's next action). The subjects then had 10 s to give input indicated by a timer running backward (“Executing in:”). The user has the options to suggest their own action (of the available actions in yellow), stop the proposed action and let the robot suggest another action (“Mach das nicht” — Do not do this) or indicate that they are indifferent about the proposed action (“Weiß ich nicht” —I do not know) and let the robot just execute it. If the subjects do not give input within the 10 s the robot executes the proposed action. **(B)** In Mode B, at first, only the upper part of the interface is active and the subjects are shown the proposed action (“Kobos nächste Action” —Kobo's next action) with pink highlighting. They have 5 s, indicated by the timer running backward (“Executing in:”), to stop the execution of the proposed action and let the robot suggest a new one (“Mach das nicht” —Do not do this). After the robot executed an action, the lower part of the interface gets activated and the subjects can give feedback about the executed action (“Feedback zur letzen Aktion” —Feedback for the last action) which can be positive (green thumbs up), negative (red thumbs down), or indifferent (“Weiß ich nicht” —I do not know). If they do not give feedback within 10 s the robot continuous to propose the next action and the upper part of the interface gets active again.

**Figure 5:**
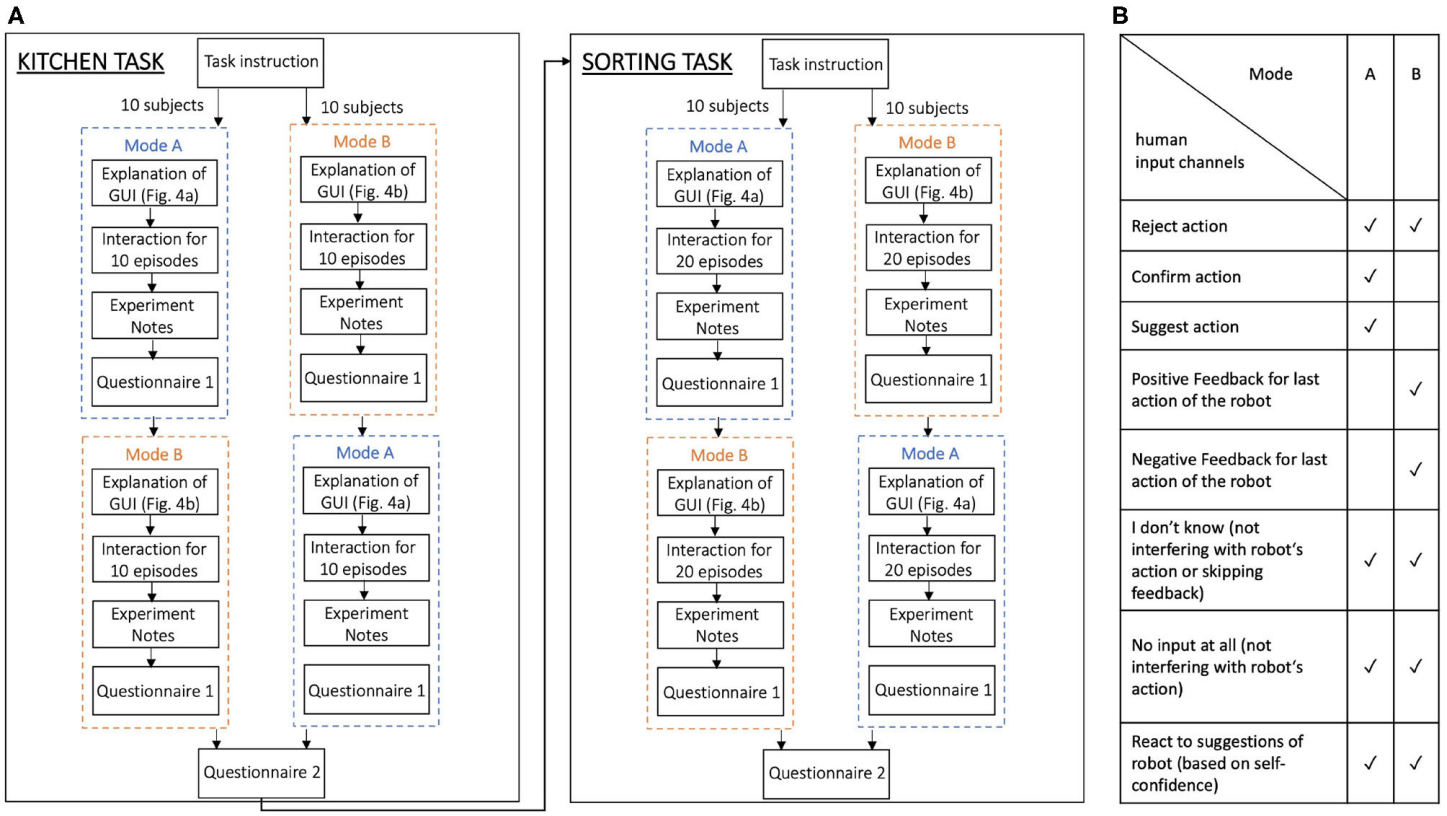
**(A)** Shows the study procedure for the evaluations with 20 subjects. In the beginning, all subjects received instructions about the kitchen task and were randomly assigned to either begin in Mode A or B. After a familiarization phase with the graphical user interface the subjects interacted with the robot for 10 episodes in the first mode and subsequently took notes on what they did and filled out Questionnaire 1. Afterward, they encountered the other mode, respectively. After the kitchen task was finished for both modes, the subjects answered Questionnaire 2, containing questions for direct comparison of Mode A and B, and then were instructed on the sorting task and repeated the same procedure on this task. However, in the sorting task, the subjects interacted for 20 episodes in each mode. **(B)** Shows the input modalities for the user's interaction with the robot that were possible in the different modes in both tasks.

We evaluate our approach on the robotic kitchen task with 20 inexperienced users. We compare two different interaction modes with our framework. In Mode A the subjects can reject actions, that are presented by the robot and can also actively suggest alternative actions. The user interface that allowed these input channels is shown in Figure 4A. In Mode B the subjects only get the options to reject actions presented by the robot and to provide feedback (positive, negative, or “I don't know”) after action execution. The interface for the input channels in Mode B is shown in Figure 4B. The table in Figure 5B summarizes and shows which input channels are possible in each mode.

Before the experiments, all subjects were introduced to the overall setting. They were told that they are supposed to help the robot to successfully complete the task at hand. In addition, they received written as well as verbal instructions that precisely explained the task goal (pour lemon juice and sugar in the bowl to complete the cocktail). We randomize the order in which Mode A and B are presented to the subjects to eliminate ordering effects. In each mode, the subjects interacted for 10 episodes with the robot. An episode is finished either by a task success, a failure, or after a maximum number of steps has been reached, which we defined as 30 in our experiments. In each mode and after each episode the participants got feedback whether the episode was finished successfully or unsuccessfully. After each episode, the initial positions of the objects were changed by the experiment supervisor, in the same order of initial locations for each subject. When a mode was completed, participants were asked to fill out experiment notes. These were blank spaces in which the subjects were asked to subjectively report how they interacted with the robot during a given mode and whether they noticed anything specific during the interaction or something stood out to them. Afterward, subjects were asked to fill out a questionnaire (Questionnaire 1) which contained questions about the participants' attitude, experience and impression of the task, the robot, and their interaction and contribution to the task. For this, participants indicated on a five-point Likert scale how much they agreed with statements about how well they thought they could communicate with the robot, how helpful they felt in its success and learning as well as whether they felt like they had control over the robot. Lastly, after completing both modes for the task, they were given a final questionnaire (Questionnaire 2) that directly compared the two modes. In it, subjects indicated in which mode they could communicate best with the robot, in which mode they felt they gave the most useful input and which mode they would prefer overall in future applications. For this, they were also able to refer back to their experiment notes so they could remember each mode correctly. This way, subjects could directly compare the modes. The steps of this study procedure are visualized in Figure 5A on the left.

**Figure 6:**
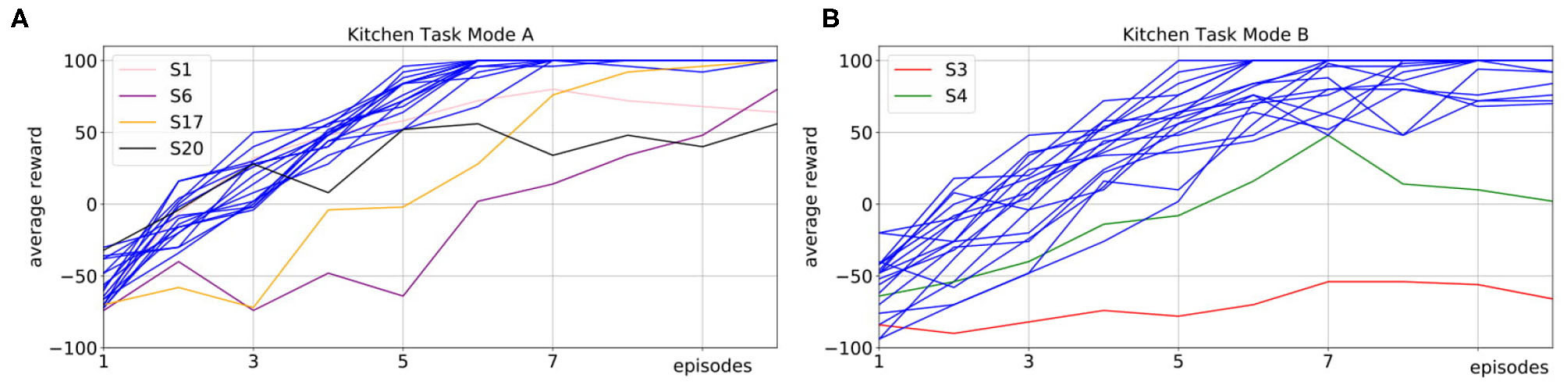
We compare two different interaction modes in experiments with 20 inexperienced subjects on the robotic kitchen task. In Mode A, subjects can prevent action execution and actively suggest own actions. In Mode B, subjects can prevent action execution and give feedback after the action execution. We show the average reward over 10 episodes of interaction, where we plot the mean over 50 evaluation runs per episode for each subject. **(A)** Shows the results for Mode A (pink, purple, orange, and black for highlighted subjects, blue for all other subjects). **(B)** Shows the results for Mode B (red, green for highlighted subjects, blue for all other subjects). The results show that in both modes, the learning speed is strongly improved compared to learning without human input (Mann-Whitney-*U* test, Mode A: *U* = 0, *z* = 6.38, *p* < 0.001, *n*_1_ = 19, *n*_2_ = 50; Mode B: *U* = 0, *z* = 6.25, *p* < 0.001, *n*_1_ = 18, *n*_2_ = 50). For most subject learning is more accelerated in Mode A as opposed to Mode B, however this difference is not significant (Wilcoxon signed-rank test, *T* = 18.5, *z* = −1.32, *p* = 0.188, *n* = 11).

Figure 6A shows the mean average rewards for all subjects in Mode A and Figure 6B shows the mean average rewards for all subjects for Mode B. To obtain a mean value of the performance after each episode, we averaged the policy taught up to that point over 50 simulated evaluations runs for each subject for each episode. The plots show that for most of the subjects the interactive learning could already reach an average maximum reward of 100 after only 10 episodes of interaction. Compared to pure RL without human interaction (Figure 3), this results in a speedup of 20 times. We investigate whether human input can significantly decrease the number of episodes it takes to reach an average reward of 80% of the maximum reward. One subject in Mode A and two subjects in Mode B never reached 80% of the maximum reward and we excluded these subjects from the following statistical analysis. For the remaining subjects it shows that compared to learning with no human input (*Mdn* = 111) in average the learning was faster in both, Mode A (*Mdn* = 5) and Mode B (*Mdn* = 5.5). A Mann-Whitney-U test shows that the differences in comparison to learning without human input are significant in both, Mode A: *U* = 0, *z* = 6.38, *p* < 0.001, *n*_1_ = 19, *n*_2_ = 50 and Mode B: *U* = 0, *z* = 6.25, *p* < 0.001, *n*_1_ = 18, *n*_2_ = 50.

When comparing Mode A and B, it shows that for nine subjects Mode A results in faster learning, for six subjects learning in both modes was equally fast and for two subjects learning in Mode A was slower than in Mode B. However, a Wilcoxon signed-rank test to evaluate if an average performance of 80% can be reached in fewer episodes in Mode A than in Mode B shows no significant difference, *T* = 18.5, *z* = −1.32, *p* = 0.188, *n* = 11.

**Figure 7:**
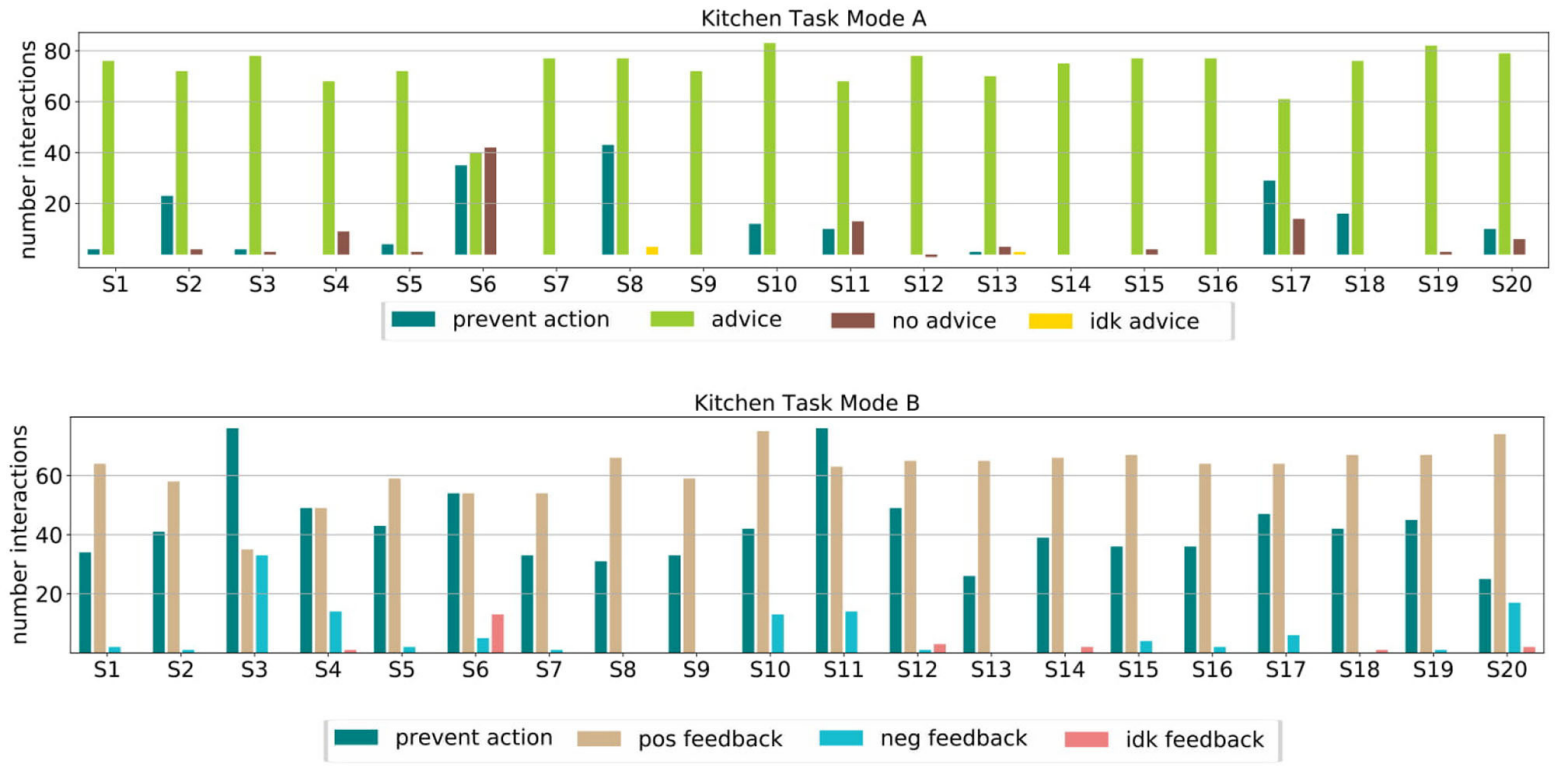
We evaluated how the subject interacted with the robot in Mode A and Mode B. The top row shows the interactions for Mode A split into the different input forms, namely preventing actions, actively suggesting actions (advice) not giving input (no advice) and the “I don't know” option (Idk advice). Most subjects actively gave advice. Notable is the behavior of subject 6, who let the robot explore a lot on its own, which resulted also in slower learning. The bottom row shows the interactions for Mode B split into the different input forms, namely preventing actions, giving positive, negative, or “I don't know” feedback. It shows that most subjects rather prevented actions than gave negative feedback. Notable is subject 3, which prevented many actions and gave a lot of negative feedback resulting in worse learning.

We highlight the subjects for which the learning did not work so well with different colors in Figure 6 to allow for connections to Figure 7. Figure 7 shows how the users interacted with the robot in Mode A (upper row) and Mode B (lower row). It shows that in Mode A most of the users actively suggest actions, except for user 6 that preferred to give less advice and just let the robot decide in most cases. This results in a slower but still steady learning process, as visualized in Figure 6A with the purple line. In Mode B the results show that the subjects use the reject option a lot, mostly in combination with positive feedback. That means most subjects used the action rejection not only to prevent possible disaster actions but to reject any action but the one they want the robot to execute and then give positive feedback for this. Subject 3 used the negative feedback in a way, that whenever the first suggestion of the robot was not correct (and needed to be changed by the subject) the subject still gave negative feedback once the correct action was chosen by the robot. As shown in Figure 6B with the red line, this resulted in problems with the learning process. In general, sometimes negative feedback was also used by the subjects to not rate the action choice but the execution of the movement, e.g., grasp position at the objects.

**Figure 8:**
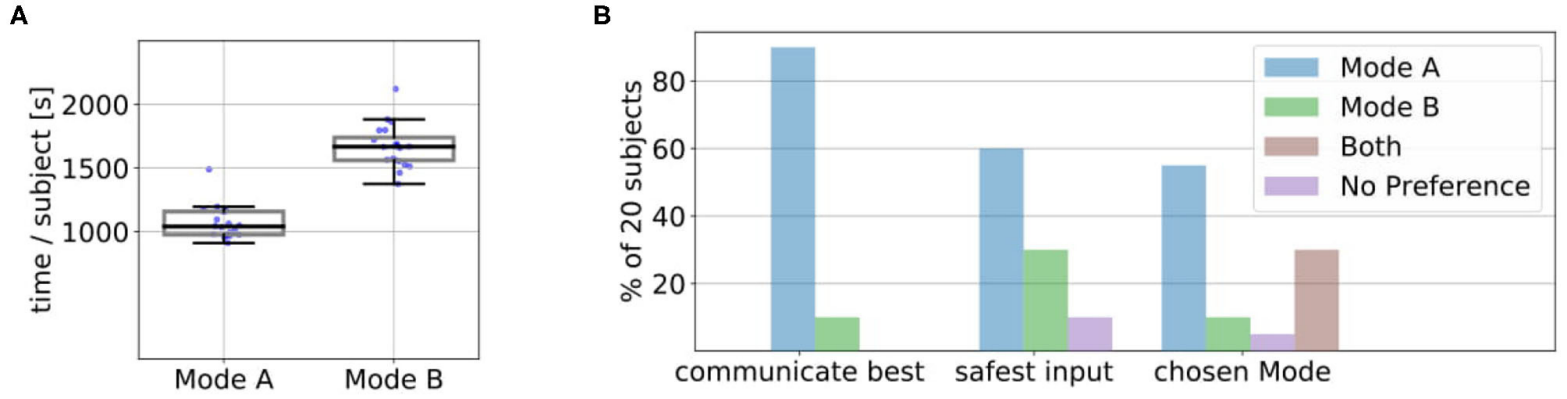
**(A)** We found that Mode A (*Mdn*=1041) was on average less time consuming than Mode B (*Mdn*=1668). A Wilcoxon signed-rank test indicated that this difference was significant, *T* = 0, *z* = −3.92, *p* < 0.001, *n* = 20. The plots show the median, interquartile range (box), 1.5 whiskers and the underlying data points. **(B)** In Questionnaire 2 the users answered subjective comparison questions about in which mode they felt they could communicate best with the robot, in which mode they felt most safe their input was useful and which mode they would choose for future interaction. In the last question, they could also choose if they prefer a combination of both modes.

Figure 8A shows the average amount of time in seconds that it took for the 10 episodes in Mode A and B over all 20 subjects and the underlying data points. We found that Mode A (*Mdn* = 1041) was on average less time consuming than Mode B (*Mdn* = 1668). A Wilcoxon signed-rank test indicated that this difference was significant, *T* = 0, *z* = −3.92, *p* < 0.001, *n* = 20. A possible reason for this could be that in Mode A the users directly suggested the actions they wanted instead of excessive rejections until the desired action was presented by the robot (Figure 7). Some users also reported in the experiment notes that they particularly preferred Mode A because of this difference in interaction time. We also noticed during the experiments that some subjects became more distracted and bored if interactions started to become slower and when they could not actively propose actions (but just passively judge or prevent them). After the experiments, the subjects answered questionnaires on how they perceived the individual modes (Questionnaire 1) and for the direct comparison of the modes (Questionnaire 2). Figure 8B shows the result of the direct comparison questions. Here a clear majority of 18 users reported they could communicate best with the robot in Mode A. When comparing, in which mode they felt safer to give useful input, most users (12 of 20) choose Mode A. Two users chose Mode B, with one user reporting in the experiment notes that Mode B required less active thinking than Mode A. For further interactions, 55% of the users would prefer to use Mode A, 10% Mode B and 30% a combination of both. Only one user found none of the modes suitable for future use. The answers to the subjective questions in Questionnaire 1 on the individual modes are shown in Figure 13. Here, a Wilcoxon signed-rank test shows that the subjects felt they controlled the robot (Q3) significantly less in Mode B than in Mode A, *T* = 18.5, *z* = −2.18, *p* = 0.029, *n* = 14. Furthermore, they indicated that they could communicate what they wanted to the robot (Q7) significantly better in Mode A compared to Mode B, *T* = 22, *z* = −2.46, *p* = 0.014, *n* = 16.

### 4.2. Robotic Sorting Task

While we assumed human input to be mostly correct and helpful in the first task, in real applications this might not always be the case. Their input might only be beneficial for solving parts of a task or if the user does not fully understand the task, their input might even be incorrect. We consider such cases to be important and introduced our concept of self-confidence in section 3.5 such that the robot is still able to learn and solve the task at hand eventually. For experimental evaluation of this concept, we designed a second robotic task to investigate how humans react in a situation when the robot starts questioning their (potentially incorrect) input.

In this second sequential robotic task, the robot is supposed to learn how to sort objects into two different boxes. In the beginning, neither the robot nor the human knows anything about the sorting criteria, but they get feedback at the end of each episode (that is sorting of a single item) about whether the episode was successful. The crucial part of this task is that the sorting criterion is defined by the weight of the objects, which can be either low (should go in box 1) or high (should go in box 2). The weights can be calculated by the robot when it lifts an object according to its joint torque sensors but the object weights are not accessible and do not get communicated to the human. However, since the objects also have different colors, as shown in Figure 2B, which do not correlate with the weights, humans could assume the objects need to be sorted by colors since this is the only accessible criterion for them. This definition of the task results in a situation where even though the human has more prior knowledge about the general structure of the task (i.e., first go to the object, then pick up the object, then bring it to a box), they have no full understanding of the task and might give incorrect input about which box to choose. If the robots questions the human's input based on its self-confidence, on the GUI a message box opens and displays the following: “I am not sure if it is a good idea to do <USER-ACTION>. I think it is better to do <ACTIONS-ACCORDING-TO-Q-FUNCTION>. Is that okay?” (Translation from German message by the authors). In case the robots Q-function had more than one optimal action the robot presents all of those in the message box and asks the user whether they want to choose one of them.

The state space of the MDP is formally defined by the weight in the robotic arm which can be EMPTY, HIGH, or LOW and its position which can be AT-HOME, AT-OBJECT, AT-BOX1, or AT-BOX2. This definition of the state space results in 12 states. The actions are defined as GO-TO-OBJECT, GO-TO-BOX1, GO-TO-BOX2, PICK-UP, and DROP which results in six actions. Not all actions are available in all states, e.g., the robot can only pick-up the object when it is close to the object and can only drop the object if he has grasped it before. The task is successfully finished if the object is dropped in the correct box. The task is finished unsuccessfully if the object is dropped in the incorrect box or is dropped when the robot is not at a box. The reward is defined as

$$\begin{array}{l} r=\{\begin{array}{l} 10\;\text{ if episode ends successful}\\ -10\text{ if episode ends not successful}\\ 0\text{ if episode ends after maximum number of steps}\\ 0\text{ in all other states that do not end the episode} \end{array} \end{array}$$

In our experiments, we used two different colors for the objects, orange and blue, however, these colors do not correlate with the weights of the objects. The scenario of the sorting task is shown in Figure 2B. In the experiments we used the heuristic increase for the self-confidence as explained in section 3.6.4, with δ_β_ = 0.2 and *I*_min_ = 7. These values where hand-tuned with respect to the average amount of episodes it took for the RL-module to learn a reasonable policy for the sorting task.

#### 4.2.1. Simulated Human Input

**Figure 9:**
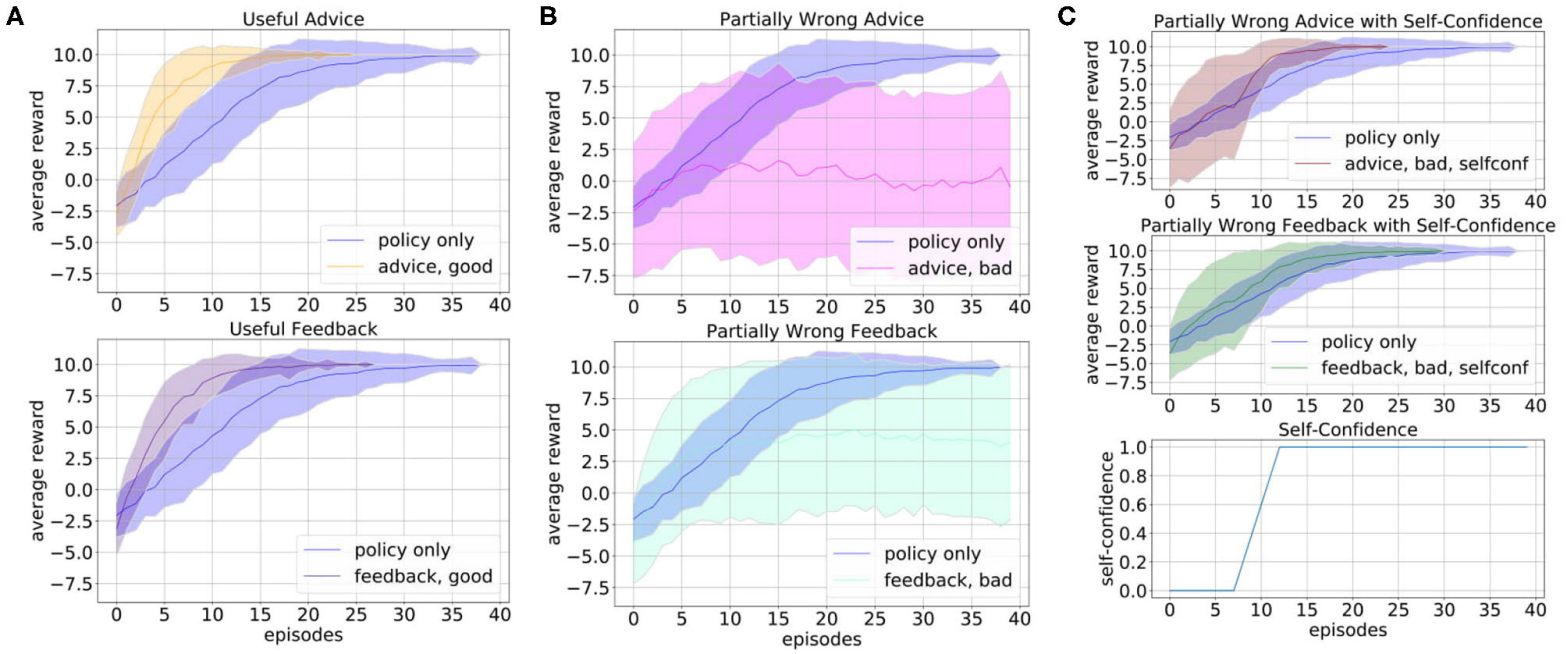
We evaluate different forms of simulated human input on the sorting task, namely action advice, and feedback after action execution. To obtain a mean value of the performance after each episode, we averaged the policy learned up to that point over 20 evaluations runs and repeated this for 100 experiments with different random seeds. The plots show the mean (solid line) and standard deviation (shaded area) of the average reward over the number of episodes. We assume that since the human does not know the correct sorting criterion the most useful input they can give is about the task structure (first go to object, then pick up the object). The positive influence of such optimal useful human input on the learning is shown in **(A)** for action advice in the top row and feedback after action execution in the bottom row. However, the human might also give suboptimal input, e.g., on which box to choose, that we simulate here by random suggestions. **(B)** shows the negative influence of this on the learning for action advice (top row) and feedback (bottom row). Using the self-confidence module **(C)** the robot becomes able to also deal with such potentially incorrect input and the learning curves for action advice (first row) and feedback (bottom row) even slightly outperform the learning without human input when using an increasing self-confidence after an initial number of training episodes (bottom row).

As for the robotic kitchen task we again first evaluate the influence of simulated human input on the learning process of the sorting task. Since for the human the sorting criterion is not obvious nor accessible we assume that there can be no correct human input on which box to choose. However, the human still has a broader picture of how “sorting” works and can provide help in structuring the task, e.g., in the beginning, the robot should always first go to the object and then pick the object up. We consider human feedback that only provides this structural information but lets the robot explore the rest of the task, i.e., which box to choose. Figure 9A shows how such useful feedback can speed up the learning. However, in this task, we can not assume that the human only gives such useful feedback but maybe also starts giving feedback on which box to choose according to his or her own hypothesis about how the sorting works. We simulate this by random input on which box to choose. Figure 9B shows that such incorrect input of the human can harm the learning process if it is not counteracted by the robot. Using the concept of self-confidence as introduced in our framework can, therefore, be beneficial in such tasks as illustrated in Figure 9C. Here, the robot after a defined number of initial training episodes stops fully trusting the human and also rejects human suggestions, with an overtime increasing probability. This results in a learning curve that is even able to slightly outperform the learning without human input for both, action advice and feedback after action execution. However, it should be noted that here we assume the human accepts all rejections of action advice, which might not always be the case with real humans.

#### 4.2.2. Real Human Input

**Figure 10:**
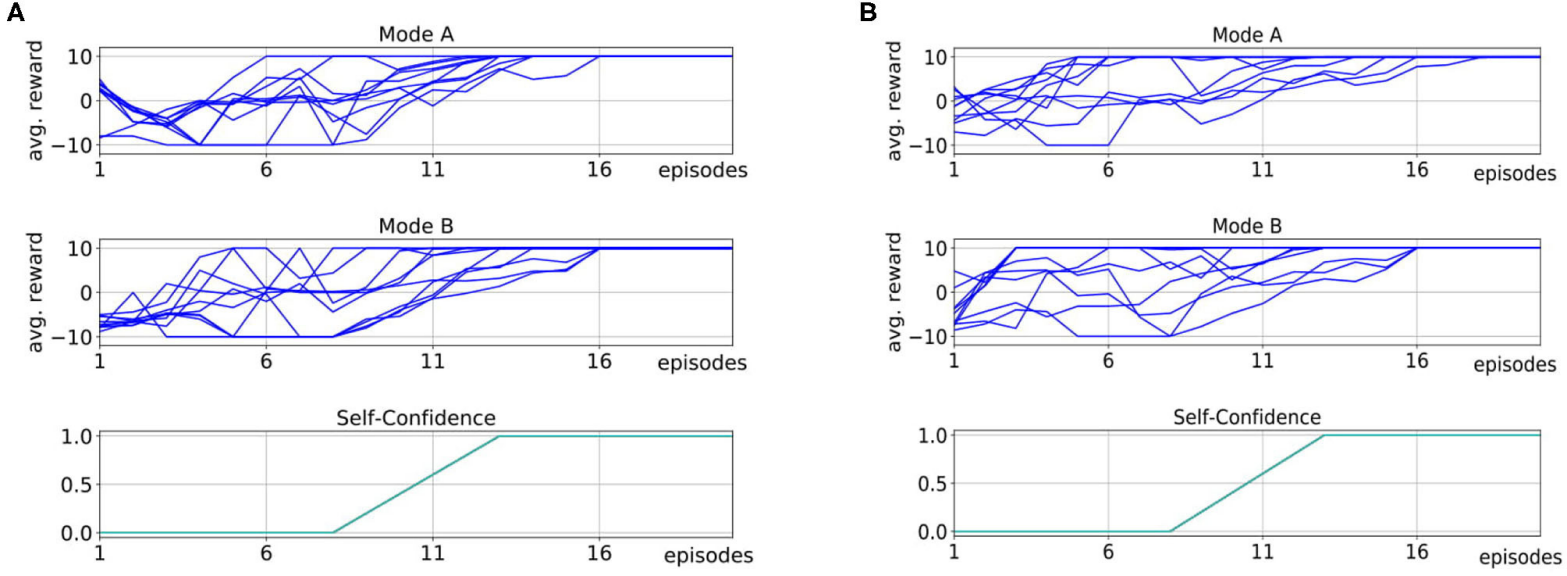
We compare the two experiment rounds **(A,B)** and the two different interaction modes across the 20 inexperienced subjects on the robotic sorting task. In Mode A (top rows) subjects can prevent action execution and actively suggest own actions. In Mode B (middle rows) subjects can prevent action execution and give feedback after the action execution. We show the average reward over 10 episodes of interaction, where we plot the mean over 50 evaluation runs per episode for each subject. The Self-Confidence is shown in the bottom row. It shows that there is no significant difference between Mode A and Mode B (Wilcoxon signed-rank test, *T* = 84.5, *z* = −0.42, *p* = 0.67, *n* = 19). However, there is a difference for some users between the first and the second round since the subjects realized between these rounds that they can not know the sorting criteria and eventually adapted to this. For all subjects, the learning could be accelerated compared to learning without human input (Mann-Whitney-*U* test, Mode A: *U* = 332.5, *z* = 4.7, *p* < 0.001, *n*_1_ = 20, *n*_2_ = 100 and Mode B: *U* = 346.5, *z* = 4.6, *p* < 0.001, *n*_1_ = 20, *n*_2_ = 100).

We evaluated our approach with the same 20 inexperienced users on the sorting task. The subjects were only told that the task goal is to sort objects correctly into the boxes. However, they did not receive any information about a sorting criterion. All subjects interacted with the robot two times (once in Mode A and once in Mode B) for 20 episodes. In each episode, one object was supposed to be sorted by the robot and at the end of an episode, the human and robot received feedback on whether the sorting was correct. We randomized the order in which the subjects faced Mode A and B to eliminate ordering effects. Overall, the study process in the sorting task, including the experiment notes and questionnaires was identical to the one described in the kitchen task and is shown in Figure 5A on the right. Figure 10 shows the average rewards of the robot's policy over the number of episodes in the first experiment (A) and the second experiment (B). To obtain a mean value of the performance after each episode, we averaged the policy taught up to that point over 50 simulated evaluations runs for each subject for each episode. The plots are also separated between the modes that the subject used in each round and show the corresponding self-confidence of the robot in the bottom row. The results show that for all subjects our approach converged to the maximum average reward in <20 episodes. Compared to the learning without human input, this is a speedup of ~25%. We investigate if human input can significantly decrease the number of episodes it takes to reach an average reward of 100% of the maximum reward. We compared learning without human input (*Mdn* = 16.5) to learning with human input for both Mode A (*Mdn* = 12) and Mode B (*Mdn* = 11). A Mann-Whitney-U test shows that the differences in comparison to learning without human input are significant in both, Mode A: *U* = 332.5, *z* = 4.7, *p* < 0.001, *n*_1_ = 20, *n*_2_ = 100 and Mode B: *U* = 346.5, *z* = 4.6, *p* < 0.001, *n*_1_ = 20, *n*_2_ = 100. However, Wilcoxon-signed rank test shows that there is no significant difference in learning speed between Mode A and Mode B, *T* = 84.5, *z* = −0.42, *p* = 0.67, *n* = 19. When comparing the results of the two rounds it shows that for some subjects the speed of learning improved in the second round, however, we think the sample size of 10 subjects is too small to perform meaningful statistical tests. When examining how the subjects gave input in the two rounds, we see that some of them changed their behavior and gave less input after they realized in the first round that they did not understand the sorting criterion. This is indicated by the average amount of input on the choice of the box subjects gave in Round 1 and Round 2. Figure 12A shows that subjects reduced (potentially incorrect) input on which box to choose in Round 2 (*Mdn* = 21) compared to Round 1 (*Mdn* = 29). In addition, on average they gave more explicit “I don't know” input for the choice of the box in Round 2 (*Mdn* = 11.5) compared to Round 1 (*Mdn* = 5). A Wilcoxon signed-rank test indicates that these differences are not significant, *T* = 53, *z* = −1.94, *p* = 0.052, *n* = 20 for input of boxes and *T* = 16.5, *z* = −1.47, *p* = 0.141, *n* = 11 for the “I don't know” input. However, since the test reveals an almost significant difference between the amount of input on which box to pick from Round 1 to Round 2 this might show that there is a shift in the users' perception on the robot's abilities and how much they trust it to choose the correct box by itself.

We also noticed in the experiments that some users would not change their behavior even if they noticed they did not understand the sorting process.

**Figure 11:**
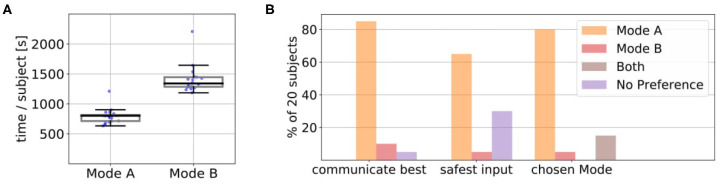
**(A)** We found that the average interaction time in Mode A (Mdn = 802.05) was on average lower than in Mode B (*Mdn* = 1340.96). A Wilcoxon signed-rank test indicated that this difference was significant, *T* = 0, *z* = −3.92, *p* < 0.001, *n* = 20. The plots show the median, interquartile range (box), 1.5 whiskers and the underlying data points. **(B)** In Questionnaire 2 the users answered subjective comparison questions about in which mode they felt they could communicate best with the robot, in which mode they felt most safe their input was useful and which mode they would choose for future interaction. In the last question, they could also choose if they prefer a combination of both modes.

Figure 11A shows the test results for total interaction times. As in the kitchen task, the total interaction time in Mode A (*Mdn* = 802.05) was on average lower than in Mode B (*Mdn* = 1340.96) and a Wilcoxon signed-rank test indicated that this difference was significant, *T* = 0, *z* = −3.92, *p* < 0.001, *n* = 20. Figure 11B shows that similar to the kitchen task in the direct comparison in Questionnaire 2 most users (16 of 20) reported that they would prefer to use Mode A for future applications while one user would use Mode B and 3 users would prefer to use a combination of both modes.

**Figure 12:**
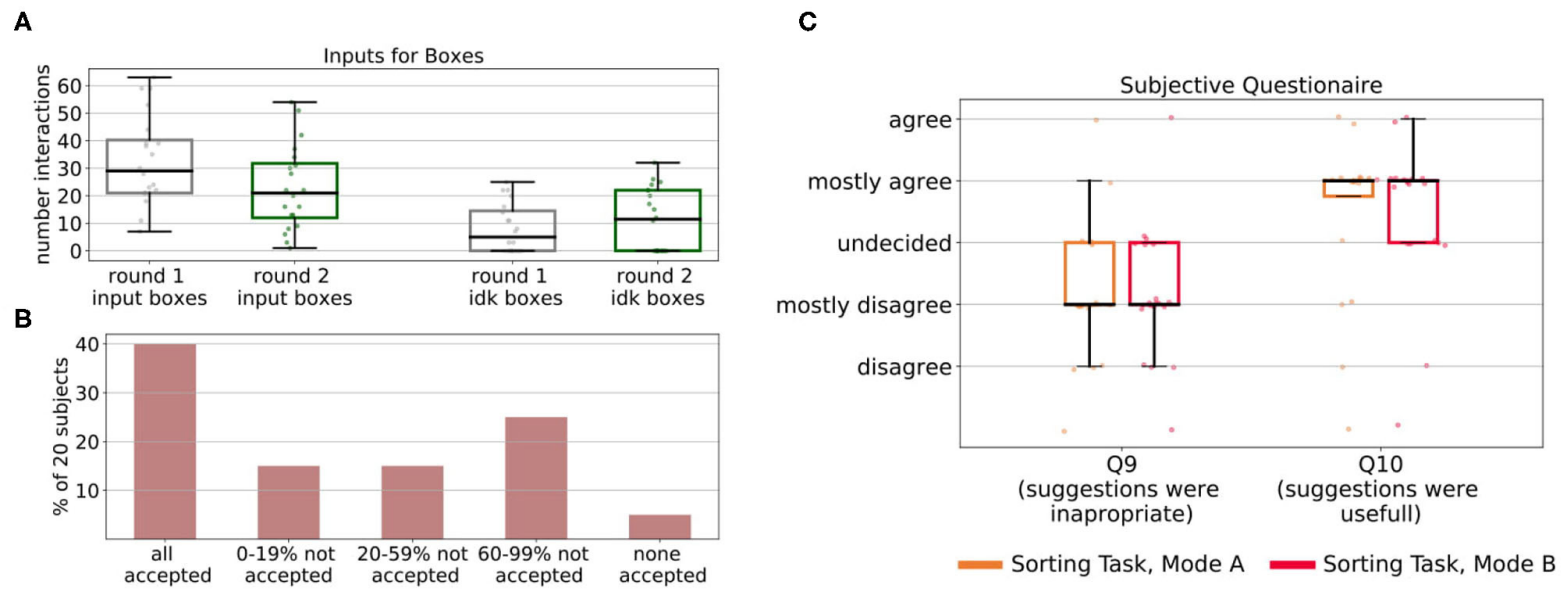
**(A)** After the first round, most users adapted their behavior and the subjects on average gave less input on which box to choose after they realized they did not understand the sorting. They also gave on average more “I don't know” input on the choice of the box. However, a Wilcoxon signed rank test indicates that these differences are not significant, *T* = 53, *z* = −1.94, *p* = 0.052, *n* = 20 for input of boxes and *T* = 16.5, *z* = −1.47, *p* = 0.141, *n* = 11 for the “I don't know” input. **(B)** Not all subjects accepted the suggestions of the robot. Only 40% of the subjects accepted all suggestions and one subject even accepted none of the suggestions. **(C)** Most subjects agreed that the suggestions were mostly useful and on average the subjects mostly disagreed that the suggestions were inappropriate.

We also evaluated how users reacted to suggestions and rejections of their input by the robot, that occurred once the robot's self-confidence started to rise. Usually, these suggestions started around episode 10. At this point, most users had already noticed that the sorting criterion was not obvious to them. However, Figure 12B shows, that only 8 out of 20 users accepted all rejections and suggestions of the robot. Some users rejected a suggestion once or twice, to see if it would have been right and afterward started to trust new suggestions and accept them. However, six users refused more than 60% of the robot's suggestions and one of them even rejected all of them. Figure 12C visualizes the answers of the users to subjective questions on the robot suggestions. It shows that while on average most subjects thought the suggestions were mostly useful and appropriate, there were also subjects that perceived them as inappropriate and not useful. A Wilcoxon signed-rank test showed no significant difference between Mode A and Mode B for inappropriateness of the suggestions (Q9), *T* = 20, *z* = −0.33, *p* = 0.739, *n* = 9, and usefulness of the suggestions (Q10), *T* = 22.5, *z* = 0, *p* = 1.0, *n* = 9. In the experiment notes, subjects reported that the robot should have given them more reasons why it suggested certain actions and explain its decisions to the users. Subjects also reported that it would have helped them to ask the robot about its abilities, e.g., whether it can see colors or if it knows the boxes weights, to understand on which basis the robot made its suggestions. Another factor that might influence the subject's perception of the robot's suggestions is that once the self-confidence rises the robot also would start to explore actions not taken before, which could sometimes seem random to the users and might created distrust in action suggestions in general. Such rejections of the robot's suggestions can cause problems if the users were able to actively suggest own actions (e.g., in Mode A), because even if the robot learned the optimal policy humans would still interfere and cause incorrect sorting of the objects.

### 4.3. Discussion

**Figure 13:**
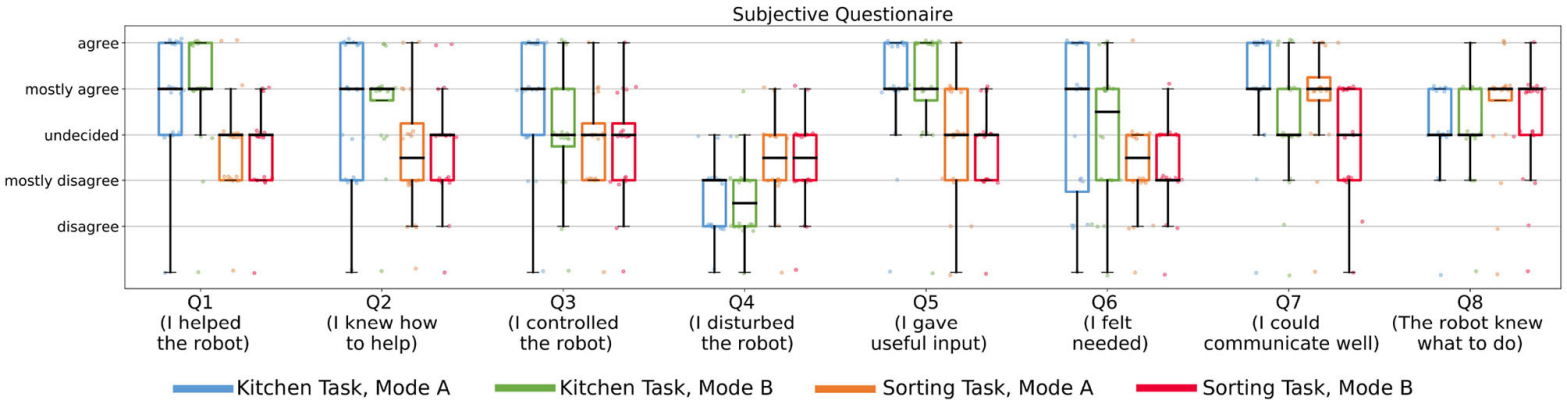
After the different modes, the subjects answered subjective questionnaires in each task on a five-point Likert scale. It shows that in the kitchen task subjects felt they controlled the robot (Q3) more in Mode A (Wilcoxon signed-rank test, *T* = 18.5, *z* = −2.18, *p* = 0.029, *n* = 14) and that they found they could communicate better (Q7) in both tasks in Mode A compared to Mode B (Wilcoxon signed-rank test, Kitchen Task: *T* = 22, *z* = −2.45, *p* = 0.014, *n* = 16; Sorting Task: *T* = 0, *z* = −2.86, *p* = 0.004, *n* = 10). Compared to the kitchen task in the sorting task the subjects felt less helpful (Q1, Wilcoxon signed-rank test, Mode A: *T* = 19.5, *z* = −2.75, *p* = 0.006, *n* = 17; Mode B: *T* = 3, *z* = −3.33, *p* = 0.001, *n* = 15) and less needed (Q6, Wilcoxon signed-rank test, Mode A: *T* = 20, *z* = −2.07, *p* = 0.038, *n* = 14; Mode B: *T* = 19.5, *z* = −2.37, *p* = 0.018, *n* = 15).

In both described tasks human input in combination with our approach accelerated the learning process in almost all cases. For both tasks, users reported at the end, when directly asked in Questionnaire 2 that they preferred giving input in Mode A compared to Mode B. We see different reasons for this. The subjects reported in the subjective questionnaire (Questionnaire 1) and their experiment notes that they could communicate their own suggestions better in Mode A (Questionnaire 1-Q7, Wilcoxon signed-rank test, kitchen task: *T* = 22, *z* = −2.45, *p* = 0.014, *n* = 16; sorting task: *T* = 0, *z* = −2.86, *p* = 0.004, *n* = 10). Compared to the kitchen task, in the sorting task the subjects felt less helpful (Questionnaire 1-Q1, Wilcoxon signed-rank test, Mode A: *T* = 19.5, *z* = −2.75, *p* = 0.006, *n* = 17; Mode B: *T* = 3, *z* = −3.33, *p* = 0.001, *n* = 15) and less needed (Questionnaire 1-Q6, Wilcoxon signed-rank test, Mode A: *T* = 20, *z* = −2.07, *p* = 0.038, *n* = 14; Mode B: *T* = 19.5, *z* = −2.37, *p* = 0.018, *n* = 15). In general, Mode A was significantly less time-consuming (Wilcoxon signed-rank test, kitchen task: *T* = 0, *z* = −3.92, *p* < 0.001, *n* = 20); sorting task *T* = 0, *z* = −3.92, *p* < 0.001, *n* = 20. We also noticed that while action suggestion was clear to most users, the concept of feedback was harder to understand. Some users would start rating how actions were executed instead of rating which action was chosen, or judge based on other factors, such as how long it took the robot to suggest the correct action as the basis for their feedback. We believe that in the future more differentiated and clear ways for feedback would be beneficial. Figure 13 shows the results of the subjective questions on the two tasks and different modes. It shows that in the kitchen task, in particular, subjects considered their input to be useful and helpful and felt more needed than in the sorting task. In general, they felt they could communicate better and controlled the robot more in Mode A than in Mode B. However, one subject reported in the experiment notes that when actively suggesting actions the robot would not really “learn” but only “replicate.” The mixed reactions of the users to suggestions of the robot or rejections of human input showed that the internal state of the robot should be more understandable to humans. It also showed that, in general, there is a difference in how users perceive the robot's suggestions which ranged from “*Me and the robot are a real team now*” to “*I feel, you want to force your opinion on me*” and “*I want an option to tell the robot don't do what you think but what I want*” (quotes translated from German by authors). Also, the answers to the questionnaires show that humans felt less needed and less useful when the robot started making own suggestions in the sorting task. We think it would be important to communicate to the users, which forms of input (e.g., on task structure) are still beneficial in such a task. This way, they would potentially feel like they contribute more to the robot's learning process. We consider these aspects of interactive learning very important for future research and applications to increase the acceptance and benefits of such interactive learning systems. We believe both tasks are easily reproducible on other robot systems and could be used as benchmark tasks for future experiments with interactive reinforcement learning systems.

## 5. Conclusion and Future Work

In this paper, we presented an approach to incorporate multiple human input channels in a reinforcement learning framework for sequential robotic tasks. Our approach also includes a concept for self-confidence such that the robot can try to reject human input after an initial training phase if it contradicts the learned policy. Experimental evaluations with 20 inexperienced users on two robotic tasks showed that human input could be incorporated beneficially to speed up the learning, even if it was partially incorrect. Direct comparison of different communication modes for the human subjects showed that most subjects preferred active participation in the learning process, e.g., through action suggestion or prohibition. However, the evaluations also showed that not all subjects would accept suggestions of the robot once the robot's self-confidence was high enough to question the human input. This was particularly prominent when they did not understand the reasons behind the robot's suggestions or the robot's learning process. We think these results align well with findings from Li et al. (2016), who report that sharing metrics, such as the robot's uncertainty with users can increase engagement during learning and with Thomaz and Breazeal (2008) who also mention the importance of communicating the robot's uncertainty to humans.

For future work, we think it is therefore important to include a more transparent communication of the robot's internal learning state into our approach. In particular, we want to investigate how communication about reasons for suggestions could help to increase acceptance of the robot's suggestions by users. The general question of how to deal with wrong human input in interactive RL systems requires further research as well. While the tasks evaluated in this paper provided already valuable insights on the interaction of humans with our interactive RL-framework, in future work we plan for additional evaluation on more realistic tasks, in particular in the context of assistant robotics, including more complex implementations for the human-advice and RL-module. To tackle more realistic and complex problems with larger state and action spaces we consider it necessary to change the current simplistic tabular representation of the advice and the RL module into more complex function approximators, with capabilities to generalize across similar states, such as a linear model of, e.g., Radial-Basis-Function-features. Moreover, we think the human advice module should be extended in a way that it also can adapt to the learning process that might happen for the human during the interaction. E.g., if the human suggests an action and then gives negative feedback after realizing unexpected effects after execution the human advice module should take this correction into account. To this end, we additionally want to consider incorporation of a way to track how recent feedback or advice on nearby states has been received as proposed in the form of their eligibility module by Knox and Stone (2012). Further, the current choice for decreasing learning and exploration rate of the RL-module should be reconsidered in future work, since even though it is a common choice in classical RL in the HRI context we see the need for adaptation to learning effects of the human and therefore the necessity to investigate different strategies of computation for learning and adaptation rates. Moreover, we consider it important to include more principled concepts for computation of the robot's self-confidence, which could also be state-dependent. One option would be to consider the convergence of the Q-function in different regions of the state space rather than just increase the self-confidence after an initial training phase as implemented for our experiments. Lastly, subjects also reported that they would have liked to communicate over different modalities besides the tablet with the robot, e.g., natural language. Also incorporating rule-based input forms or options to teach sequences of coupled actions were suggested by the users and could be potentially incorporated in our approach in future work.

## Data Availability Statement

The datasets generated for this study are available on request to the corresponding author.

## Ethics Statement

Written informed consent was obtained from the individual(s) for the publication of any potentially identifiable images or data included in this article.

## Author Contributions

DK contributed by writing the manuscript, developing the proposed approach, coding, planning, preparing, and executing the experiments. MK contributed to the development of the proposed approach. VS contributed to the planning and executing the experiments and the writing of the manuscript. CD'E contributed to the development of the proposed approach and the writing of the manuscript. JP contributed to the writing of the manuscript. All authors contributed to the article and approved the submitted version.

## Conflict of Interest

The authors declare that the research was conducted in the absence of any commercial or financial relationships that could be construed as a potential conflict of interest.
